# Repeated measures ASCA+ for analysis of longitudinal intervention studies with multivariate outcome data

**DOI:** 10.1371/journal.pcbi.1009585

**Published:** 2021-11-09

**Authors:** Torfinn S. Madssen, Guro F. Giskeødegård, Age K. Smilde, Johan A. Westerhuis

**Affiliations:** 1 Department of Circulation and Medical Imaging, Norwegian University of Science and Technology, Trondheim, Norway; 2 Biosystems Data Analysis Group, Swammerdam Institute for Life Sciences, University of Amsterdam, Amsterdam, The Netherlands; Universitat Heidelberg Zentrum fur Molekulare Biologie, GERMANY

## Abstract

Longitudinal intervention studies with repeated measurements over time are an important type of experimental design in biomedical research. Due to the advent of “omics”-sciences (genomics, transcriptomics, proteomics, metabolomics), longitudinal studies generate increasingly multivariate outcome data. Analysis of such data must take both the longitudinal intervention structure and multivariate nature of the data into account. The ASCA+-framework combines general linear models with principal component analysis and can be used to separate and visualize the multivariate effect of different experimental factors. However, this methodology has not yet been developed for the more complex designs often found in longitudinal intervention studies, which may be unbalanced, involve randomized interventions, and have substantial missing data. Here we describe a new methodology, repeated measures ASCA+ (RM-ASCA+), and show how it can be used to model metabolic changes over time, and compare metabolic changes between groups, in both randomized and non-randomized intervention studies. Tools for both visualization and model validation are discussed. This approach can facilitate easier interpretation of data from longitudinal clinical trials with multivariate outcomes.

## Author summary

Clinical trials are increasingly generating large amounts of complex biological data. Examples can include measuring metabolism or gene expression in tissue or blood sampled repeatedly over the course of a treatment. In such cases, one might wish to compare changes in not one, but hundreds, or thousands of variables simultaneously. In order to effectively analyze such data, both the study design and the multivariate nature of the data should be considered during data analysis. ANOVA simultaneous component analysis+ (ASCA+) is a statistical method which combines general linear models with principal component analysis, and provides a way to separate and visualize the effects of different factors on complex biological data. In this work, we describe how repeated measures linear mixed models, a class of models commonly used when analyzing changes over time and treatment effects in longitudinal studies, can be used together with ASCA+ for analyzing clinical trials in a novel method called repeated measures-ASCA+ (RM-ASCA+).

This is a *PLOS Computational Biology* Methods paper.

## Introduction

In recent decades, scientific and technological developments have increased our ability to both collect and manage large amounts of data. In biomedicine this has contributed to the rise of various “-omics”-fields (e.g. genomics, transcriptomics, proteomics, and metabolomics), where the focus is not on single variables such as individual genes, proteins or metabolites, but rather on the whole genome, proteome, or metabolome. Because “-omics”-datasets may contain hundreds to thousands of variables, considering each variable individually may be inefficient. In addition, “-omics”-data are multivariate by nature, and performing separate analyses for each variable does not take the interrelatedness between the variables into consideration.

A subset of the methods developed to analyze such data are based on the idea of combining two different kinds of statistical methods: 1) analysis of variance (ANOVA), and 2) dimensionality reduction methods such as principal component analysis (PCA) [1]. Combining these methods in various ways allows the researcher to separate and visualize effects from different sources of variation in the data, while also accounting for the correlations between the outcome variables. One such method is ANOVA simultaneous component analysis (ASCA), where the response matrix is first decomposed into effect matrices according to the experimental design, and the impact of each experimental factor is visualized by applying PCA to the effect matrices [2]. This methodology has since been extended to unbalanced designs by the adoption of the general linear model (GLM)-framework in estimating the effects, in a method called ASCA+ [3]. A related approach was recently developed, termed linear mixed model-PCA (LiMM-PCA), in which the ASCA+ methodology is adapted to include random effects [4].

Longitudinal intervention studies with repeated measurements over time are an important type of experimental design in biomedical research. Such designs allow separating within-subject variation from between-subject variation, and also permit the study of trends over time. However, repeated measures data have properties which preclude the use of standard linear regression methods, such as fixed effects ANOVA, the most important one being that the observations belonging to the same individual are not independent. While the classical ASCA methodology has long been applied for longitudinal data analysis [2,5], this approach has generally required strongly balanced designs to yield valid results. In contrast, longitudinal intervention studies routinely have many complicating features, such as unbalanced designs, randomized interventions, and substantial missing data. Repeated measures linear mixed models provide a powerful way to handle these issues [6], but these capabilities have so far not been extended to the multivariate case.

In this paper we introduce a new methodology, repeated measures ASCA+ (RM-ASCA+), using repeated measures linear mixed models in conjunction with ASCA+. We show how this method can be used in the analysis of longitudinal multivariate data with unbalanced designs and missing outcome data, including both visualization of results and assessment of model uncertainty. We also discuss other linear models for longitudinal data which can be used to estimate the effects, and comment on their suitability for ASCA+. We discuss how to adjust the analyses depending on whether the intervention is randomized or not, and we illustrate these differences by analyzing two different metabolomics datasets. We compare our findings with the original research papers for these datasets, and discuss the added benefit of RM-ASCA+ for biological interpretation in this setting.

## Methods

### Linear models for longitudinal data

A key step in ASCA+ is to define an appropriate linear model to use for estimating the effects. In this section we discuss some possible model types for analysis of longitudinal repeated measures data, and comment on their suitability for ASCA+. These models differ both in how they handle missing data, and whether they control for the baseline value of the response variable. The latter point can strongly affect the effect estimates if the groups come from different pre-baseline populations, which is known as Lord’s paradox [7]. The models to be discussed are: 1) repeated measures models, 2) longitudinal analysis of covariance (ANCOVA), and 3) analysis of changes. All model types discussed here involve including a random intercept for each subject, except for longitudinal ANCOVA and analysis of changes in the case of only two measurement occasions, as in this setting each subject only appears once in the response vector, and there is no need to account for within-subject correlation. The linear models presented here are the same as presented in the paper by Twisk et al., concerning different ways of analyzing randomized controlled trials with repeated measurements [8].

#### Repeated measures

In a repeated measures model, the baseline value of the response variable is included in the responses in the same way as the follow-up measurements. Suppose that a response variable *y* is measured at *K* timepoints (*k* = 1..*K*) for a total of *I* subjects (*i* = 1..*I*), where each subject belongs to one of *H* groups (*h* = 1..*H*). If the number of timepoints *K* = 3, and the number of groups *H* = 2, then a repeated measures model for this data is:

$$y_{ihk}=(\beta_{0}+\gamma_{0i})+\beta_{1}t_{1k}+\beta_{2}t_{2k}+\beta_{3}g_{h}+\beta_{4}(t_{1k}*g_{h})+\beta_{5}(t_{2k}*g_{h})+e_{ihk}$$

where *β*_*0–5*_ are the coefficients to be estimated from the data, *t*_*1k*_ and *t*_*2k*_ are indicator values for the time factor, *g*_*h*_ is an indicator variable value for the group factor, *t*_*1k*_ * g_*h*_ and *t*_*2k*_ * g_*h*_ are factor interaction values, *γ*_*0i*_ represents a subject-specific random intercept, which is $∼N(0,\sigma_{u}^{2})$, and *e*_*ihk*_ is a residual term, which is $∼N(0,\sigma_{e}^{2})$. Both the random intercepts and residuals are assumed to be independent. The model is here shown for only three timepoints and two groups, but can easily be extended to an arbitrary number of timepoints and groups by including more indicator variables.

When entering categorical variables (e.g. experimental factors such as time and treatment) into a regression model, a choice must be made regarding how the different factor levels should be represented in the model. In general, to enter a categorical factor with *k* levels, (*k*– 1) variables are needed. While choice of coding does not impact the overall fit of the model, it determines the interpretation of the model coefficients, and therefore also the interpretation of the effect matrices estimated by ASCA. Two commonly used coding systems for representing categorical variables are reference coding and sum coding. With reference coding, one of the factor levels is selected as the reference level, and represented by setting all indicator variables to zero, while the other levels are represented by one of the indicator variables taking on the value 1. Thus, representing the time factor with reference coding can be done as:

$$\begin{array}{ccc} & t_{1} & t_{2}\\ k=1 & 0 & 0\\ k=2 & 1 & 0\\ k=3 & 0 & 1 \end{array}$$

This coding causes the effects of *t*_*1*_ and *t*_*2*_ to be expressed relative to the baseline timepoint *t*_*0*_ (*k* = 1, the reference level). In sum coding no reference level is selected, but every level is instead compared to the mean across all levels. To represent the time factor with sum coding, instead of representing the omitted level with a row of zeros, it is represented by a row of -1:

$$\begin{array}{ccc} & t_{1} & t_{2}\\ k=1 & -1 & -1\\ k=2 & 1 & 0\\ k=3 & 0 & 1 \end{array}$$

With this coding, the effects of *t*_*1*_ and *t*_*2*_ are expressed relative to the mean response across all three levels. The effect of *t*_*0*_, or whichever level is omitted, is not directly estimated, but can be calculated from the remaining effects. In the models discussed in this section, the indicator variables for time (*t*_*1*_ and *t*_*2*_) will be reference coded with the first timepoint as the reference level. Depending on whether the indicator variable *g* is reference coded (0 or 1) or sum coded (-1 or 1), the coefficients for time, *β*_*1*_ and *β*_*2*_ in the repeated measures model will represent either the time effect (i.e. change from baseline) for the reference group, or the average time effect across both groups. In both cases *β*_*3*_ represents the group differences at baseline, while the interaction effects *β*_*4*_ and *β*_*5*_ represent the group difference in within-group change from baseline to each of the timepoints. If *g* is reference coded, the intercept (*β*_*0*_) represents the estimated baseline mean for the reference group, while if *g* is sum coded, it represents the baseline mean across both groups.

When assessing whether the mean change in the response variable over time is different between the groups, a decision has to be made whether an adjustment for baseline differences in the response variable should be made. Although it is often believed that such an adjustment is made by assessing changes instead of directly comparing means, this is not correct (8). This is in part because the group with the highest baseline value will be expected to decrease slightly more than the group with the lowest baseline value, due to regression to the mean, even if the treatment has no effect. Conversely, the group with the lowest baseline value will be expected to increase slightly more. Simply comparing changes without adjusting for this variation can therefore lead to either over- or underestimation of the true treatment effect. The interaction coefficients *β*_*4*_ and *β*_*5*_ in the above model are unadjusted, because they are only assessing whether the within-group change is different between the groups. Typically, one adjusts for a variable by including it as a covariate. However, in a repeated measures model, where the baseline values already are included in the responses, an adjustment can instead be made by removing the main effect for treatment, *β*_*3*_*g*_*h*_, from the model while keeping its interactions with time:

$$y_{ihk}=(\beta_{0}+\gamma_{0i})+\beta_{1}t_{1k}+\beta_{2}t_{2k}+\beta_{3}(t_{1k}*g_{h})+\beta_{4}(t_{2k}*g_{h})+e_{ihk}$$

Because the time factor is reference coded with baseline as the reference level, and because there is no main effect for treatment, the estimated group means are constrained to be the same at baseline. This is also known as a constrained longitudinal data analysis (cLDA) model [9], while the previous model with the main effect for treatment included is sometimes referred to as an unconstrained longitudinal data analysis (ucLDA) model. The result of this constraint is that the interaction effects *β*_*3*_ and *β*_*4*_ will be adjusted for baseline.

In addition to adjusting for the baseline value of the response variable, it is also possible to adjust for other baseline covariates. In general, the decision of whether to adjust for baseline covariates depends primarily on the study design, and the research question of interest. When using general linear (mixed) models to analyze treatment effects in a randomized controlled trial, and the treatment effect is expressed as a difference in means, it is generally recommended to adjust for baseline (pre-randomization) covariates which are known in advance to influence the response variable [10–12]. Because of the randomization, doing so does not bias or change the interpretation of the treatment effect, but results in smaller standard errors, and thereby increased precision [13]. This property of the treatment effect estimator is referred to as collapsibility, meaning that the marginal and conditional treatment effect estimates are on expectation equal in the absence of confounding. Another situation where covariate adjustment can increase statistical power is in trials with stratified randomization. This is because the stratification induces a positive correlation between the treatment groups, which results in too wide confidence intervals for the treatment effect estimate. This can be accounted for by including the stratification factor as a covariate in the model [14,15]. These possibilities have so far not been leveraged in ASCA, but this can be done in our framework.

For non-randomized studies, the situation is more complex. In this situation, the treatment groups are typically already different before treatment is given. Adjusting for baseline covariates in this setting can induce spurious effects, except in situations in which the treatment allocation is determined by the included baseline covariates (e.g. regression discontinuity designs) [10]. The adjusted estimate will then have a different interpretation from the unadjusted one. This phenomenon is known as Lord’s paradox [16], and implies that baseline adjustments must be done with care in non-randomized settings. In general, the decision to adjust for any covariate is dependent on prior knowledge about the study design and the measured variables, as well as on assumptions about how they causally interact. This applies to all forms of covariate adjustment, of which baseline adjustment is only a special case. Causal diagrams in the form of directed acyclic graphs provide a general framework for determining whether adjustment for a given variable creates or reduces bias with respect to the effect of interest [17].

In our examples we model time as a categorical variable, as is typical in ASCA. However, time can also be modeled as a continuous variable, where different functional forms can be assumed for the time effect, e.g. linear, polynomial, or splines [18,19]. Additionally, more complex random effect structures can be considered, for example modeling time with random slopes. However, we will limit our discussion to random intercept models, in order to keep the number of estimated parameters to a minimum.

#### Longitudinal ANCOVA

The method of longitudinal ANCOVA involves using the baseline value, *y*_*ih1*_, as a covariate, instead of modeling it as a response together with the follow-up values. When *K* = 3, and *H* = 2, this model can be written as:

$$y_{ihk}=(\beta_{0}+\gamma_{0i})+\beta_{1}t_{k}+\beta_{2}g_{h}+\beta_{3}(t_{k}*g_{h})+\beta_{4}y_{ih1}+e_{ihk}$$

In this example, because the first timepoint is not included in the response vector, only two timepoints are represented in *y*, which can be described by one indicator variable *t*. In this model *β*_*2*_ represents the treatment effect at the first follow-up timepoint, while (*β*_*2*_ + *β*_*3*_) represents the treatment effect at the second follow-up timepoint, and *β*_*4*_ represents the effect of the baseline value on the follow-up measurements. The effects *β*_*0*_ and (*β*_*0*_ + *β*_*1*_) represent the expected change from baseline to the first and second follow-up timepoints, respectively, for a subject for which the baseline covariate is equal to zero. Since this is often not biologically meaningful, especially if the baseline covariate cannot plausibly take on this value in reality, it is sometimes recommended to baseline-center both the responses and the baseline covariates [20]. If this is done, then *β*_*0*_ and (*β*_*0*_ + *β*_*1*_) can be interpreted as the expected change from baseline to the first and second follow-up for the reference group, which is the same interpretation as *β*_*1*_ and *β*_*2*_, respectively, in the repeated measures model.

Like the repeated measures model, this model can be extended to more timepoints by increasing the number of indicator variables for time and its interaction with group. Clearly, longitudinal ANCOVA involves an adjustment for baseline, because it is included as a covariate in the model. It can be shown that cLDA and longitudinal ANCOVA are mathematically related. Differences in point estimates and confidence intervals for the group effect disappear with increasing sample size under randomization, and are usually small for non-randomized data [11]. However, a disadvantage of longitudinal ANCOVA compared with cLDA is that subjects with missing baseline data are excluded from the analysis.

#### Analysis of changes

Analysis of changes involves expressing all follow-up values as differences from baseline, without including the baseline response as either a response or covariate in the model. A linear model, or mixed model if there is more than one follow-up measurement, is then made to assess whether the average (*y*_*ihk*_−*y*_*ih*1_) differs significantly depending on group and time:

$$y_{ihk}-y_{ih1}=(\beta_{0}+\gamma_{0i})+\beta_{1}t_{k}+\beta_{2}g_{h}+\beta_{3}(t_{k}*g_{h})+e_{ihk}$$

Testing *β*_*2*_ and (*β*_*2*_ + *β*_*3*_) in this model effectively amounts to testing the same null hypotheses as *β*_*4*_ and *β*_*5*_, respectively, in the ucLDA-model, namely whether the time effect, or equivalently, the within-group change, is the same in both groups [11]. Similarly to longitudinal ANCOVA with baseline-centering, the coefficients *β*_*0*_ and (*β*_*0*_ + *β*_*1*_) represent the expected change from baseline to the first and second follow-up timepoints, respectively, for the reference group. While all available response data is used in ucLDA, analysis of changes excludes responses where either the baseline or follow-up measurement is missing. As with ucLDA, no baseline adjustment is made in this model. If the baseline value is added as a covariate, then the model becomes equivalent to a longitudinal ANCOVA (8). Analysis of changes shares the previously mentioned disadvantages as longitudinal ANCOVA, such as not including the baseline measurements in the response vector, and poorer efficiency in the presence of missing outcome data.

### ASCA^+^ / LiMM-PCA

#### ASCA

Suppose we have measured *J* (*j* = 1..*J*) response variables for *I* (*i* = 1..*I*) subjects at *K* (*k* = 1..*K*) measurement occasions. The responses are collected in a *IK*×*J* response matrix *Y*. In classical ASCA, the response matrix *Y* is decomposed into effect matrices according to the experimental design with standard ANOVA calculations, using differences in level averages:

$$Y=M_{\mu }+M_{T}+M_{G}+M_{T*G}+E$$

where *M*_*μ*_ is an overall offset matrix, *M*_*T*_ contains the estimated level averages for the time factor, *M*_*G*_ contains the estimated level averages for the group main effect, *M*_T*G_ contains the level averages for the interaction between time and group, and *E* is the residual matrix. Scaling can also be applied to the columns of *Y* before estimating the effect matrices. The type of scaling will usually depend on the type of data being analyzed, and which effect is being investigated [21]. The effect matrices can then be analyzed and interpreted using PCA:

$$\begin{array}{c} Y=M_{\mu }+T_{T}P_{T}\prime +T_{G}P_{G}\prime +T_{T*G}P_{T*G}\prime +E \end{array}$$

For each fixed effect *f* where *f*∈{*T*, *G*, T*G}, *T*_*f*_ is an *IK*×*A*_*f*_ score matrix, and *P*_*f*_ is a *J*×*A*_*f*_ loading matrix, where *A*_*f*_ is the number of principal components needed to describe *M*_*f*_. The symbol ′ indicates the matrix transpose. Because the effects estimated in classical ASCA are expressed relative to the overall mean, the effect matrices are implicitly mean-centered. However, in later sections in this paper we will also apply a bootstrapping step in the analysis, which involves re-centering the matrices before PCA. For this reason, we will always apply mean-centering before PCA in the following sections.

By applying PCA to the effect matrices, as opposed to the full response matrix *Y*, each multivariate sub-model will be optimal for describing the variation contributed by that factor. However, this method is limited in that it only allows inclusion of fixed effects, and that classical ANOVA effect estimators based on differences in level means results in biased effect estimates for unbalanced designs. Classical ASCA is therefore only valid for longitudinal studies if the design is strongly balanced, and with fixed effects only.

#### ASCA+

In ASCA+, the original ASCA-methodology is extended to unbalanced designs by using general linear models (GLM) to estimate the effect matrices, instead of the classical ANOVA estimators based on differences in means. The GLM can be written as:

y=Xβ+e

Where *y* is an *IK*×1 vector containing the responses, *X* is an *IK*×*p* design matrix for the chosen linear model, where *p* is the number of fixed effect predictors, *β* is a *p*×1 vector of coefficients to be estimated from the data, and *e* is an *IK*×1 vector containing the residuals. In ASCA+, this equation is extended to multivariate responses:

Y=XB+E

where *Y* now represents an *IK*×*J* response matrix, where *J* (*j* = 1..*J*) is the number of response variables, *B* is a *p*×*J* parameter matrix, where the *j*-th column of *B* corresponds to the regression coefficients belonging to the *j*-th column of *Y*, and *E* is a *IK*×*J* residual matrix. To estimate *B*, a GLM based on the design matrix *X* is applied to every column of *Y* separately, and the coefficients are collected in the matrix *B*.

After estimation of *B*, the effect matrices are then made by multiplying the corresponding columns of *X* together with the corresponding rows of *B*. For example, in order to make the effect matrix for the time effect, all columns in *X* and rows in *B* except those belonging to the time factor are turned into zero, and the following operation is done to produce the effect matrix for time, *M*_*T*_:

$$\left(\begin{array}{cccccc} Int & T_{1} & T_{2} & G & T_{1}G & T_{2}G\\ 0 & -1 & -1 & 0 & 0 & 0\\ 0 & 1 & 0 & 0 & 0 & 0\\ 0 & 0 & 1 & 0 & 0 & 0\\ 0 & -1 & -1 & 0 & 0 & 0\\ 0 & 1 & 0 & 0 & 0 & 0\\ 0 & 0 & 1 & 0 & 0 & 0\\ \vdots & \vdots & \vdots & \vdots & \vdots & \vdots \end{array}\right)\times \left(\begin{array}{ccc} 0 & \cdots & 0\\ b_{11} & \cdots & b_{1J}\\ b_{21} & \cdots & b_{2J}\\ 0 & \cdots & 0\\ 0 & \cdots & 0\\ 0 & \cdots & 0 \end{array}\right)=M_{T}$$

The effect matrix *M*_*T*_, which now contains the estimated level averages for the factor for time, can be analyzed by PCA in order to visualize the multivariate differences between the timepoints.

In ASCA+, all fixed effects are encoded using sum coding. This causes all main effects to be expressed relative to the grand mean, and all fixed effects to sum to zero. This coding ensures that the estimated effect matrices are orthogonal to each other in balanced designs. Because of this, we can quantify the unique variance contribution from each factor as:

$${\left\|Y\right\|}^{2}={\left\|M_{\mu }\right\|}^{2}+{\left\|M_{T}\right\|}^{2}+{\left\|M_{G}\right\|}^{2}+{\left\|M_{T*G}\right\|}^{2}+{\left\|E\right\|}^{2}$$

where ‖*M*‖^2^ denotes the squared Frobenius norm of the matrix *M*. This allows quantification of how much of the total variation is explained by each of the factors in the model [1].

#### LiMM-PCA

In LiMM-PCA, the ASCA+ methodology is further adapted to also include random effects, making it a potentially suitable method for correctly modeling the longitudinal structure of intervention studies. In LiMM-PCA, the response matrix *Y* is first reduced and orthogonalized by PCA, so that *T*_*A*_ is used instead of *Y* directly:

$$\begin{array}{c} Y=T_{A}P_{A}\prime +E_{A} \end{array}$$

where *T*_*A*_ is an *IK*×*A* score matrix, *A* is the number of included components in the model, and *P*_*A*_ is a *J*×*A* loading matrix. The residual matrix *E*_*A*_ is assumed to be negligible. The score matrix *T*_*A*_ is then analyzed following the ASCA+ methodology, but using mixed models instead of GLMs.

$$\begin{array}{c} T_{A}=XB+ZU+E \end{array}$$

where *B* now is a *p*×*A* fixed effect coefficient matrix, *Z* is an *IK*×*R* design matrix for the random effects, where *R* is the number of random effect coefficients for one response variable, and *U* is an *R*×*A* matrix containing all the random effect coefficients. The matrix *T*_*A*_ is then decomposed into effect matrices for the fixed effects (*f* = 1..*F*) and random effects (*r* = 1..*R*) as:

$$\begin{array}{c} T_{A}=M_{\mu }+\sum_{f=1}^{F}M_{f}+\sum_{r=1}^{R}M_{r}+E \end{array}$$

Each effect matrix can now be analyzed with PCA, as shown previously. However, because of the initial orthogonalization of the response variables, the loadings for each sub-model have to be transformed back into the original variable space of *Y* before interpretation. As long as *A* is set sufficiently high so that all of the variation in *Y* was included during the initial PCA-step, this will result in score- and loading plots close or identical to what would have been obtained if the procedure was applied directly to *Y*.

In LiMM-PCA the statistical significance for a fixed or random effect *g* is assessed using a multivariate extension of the log likelihood ratio test, which is here briefly described. First the likelihood ratio is calculated for each of the column vectors in *T*_*A*_. For fixed effects, maximum likelihood must be used. Because the columns vectors in *T*_*A*_ are orthogonal, these likelihood ratios may be added together into a global log likelihood ratio test statistic:

$${GLLR}_{g}test\;statistic=2[\sum_{a=1}^{A}\left(log(Likelihood_{\left(H_{1}a\right)})-log(Likelihood_{\left(H_{0}a\right)}))\right]$$

where $Likelihood_{\left(H_{1}a\right)}$ denotes the likelihood of the full model as calculated for the *a*-th column vector in *T*_*A*_, and $Likelihood_{\left(H_{0}a\right)}$ denotes the likelihood of the model without the effect(s) *g*. A null hypothesis distribution for the GLLR-statistic is simulated using parametric bootstrapping, which is compared with the observed GLLR-statistic to calculate a p-value.

#### RM-ASCA+

In the methodology presented here, RM-ASCA+, we combine repeated measures linear mixed models with ASCA+ to estimate the multivariate effects of time and the interaction between time and group in an unbalanced setting, while also accounting for within-subject dependency. We do this without applying the initial PCA-step as done in LiMM-PCA, and the effects are therefore estimated directly based on the full response matrix:

Y=XB+ZU+E

where *B* is a *p*×*J* fixed effect parameter matrix, and *U* is an *IK*×*J* matrix containing the random effect coefficients, and *E* is an *IK*×*J* residual matrix. Since all of the variation in *Y* is included when estimating the effects, it avoids the issue of selecting the appropriate number of components as in LiMM-PCA, although the methods for hypothesis testing and quantifying explained variance are also lost as a result. The effect matrices are then constructed in the same way as in ASCA+/LiMM-PCA:

$$Y=M_{\mu }+\sum_{f=1}^{F}M_{f}+\sum_{r=1}^{R}M_{r}+E$$

However, in order to obtain the baseline constraint as shown previously, it is necessary to deviate from the sum coding usually used in ASCA+. This is because constraining the baseline means requires the time factor to be reference coded with baseline as the reference timepoint, as was shown in cLDA. For illustration, suppose we fit a RM-ASCA+ model using an ucLDA-model, where the time effect is reference coded with baseline as reference, and group is sum coded:

$$\left(\begin{array}{cccccc} Int & T_{1} & T_{2} & G & T_{1}G & T_{2}G\\ 1 & 0 & 0 & -1 & 0 & 0\\ 1 & 1 & 0 & -1 & -1 & 0\\ 1 & 0 & 1 & -1 & 0 & -1\\ 1 & 0 & 0 & 1 & 0 & 0\\ 1 & 1 & 0 & 1 & 1 & 0\\ 1 & 0 & 1 & 1 & 0 & 1\\ \vdots & \vdots & \vdots & \vdots & \vdots & \vdots \end{array}\right)\times \left(\begin{array}{ccc} b_{01} & \cdots & b_{0J}\\ b_{11} & \cdots & b_{1J}\\ b_{21} & \cdots & b_{2J}\\ b_{31} & \cdots & b_{3J}\\ b_{41} & \cdots & b_{4J}\\ b_{51} & \cdots & b_{5J} \end{array}\right)={M_{0}+M}_{T}+M_{G}+M_{T*G}$$

With this coding system the intercept matrix *M*_*0*_ represents the overall baseline mean, while *M*_*T*_ represents the time effect expressed as the change from *M*_*0*_. The matrix *M*_*G*_ represents the group differences at baseline, expressed as deviations from *M*_*0*_, while *M*_T*G_ represents the group difference in within-group change (i.e. the treatment effect) from baseline to each of the timepoints, expressed as deviations from the general time effect.

As discussed, the treatment effect estimated in a ucLDA-model is not adjusted for baseline. If the treatment effect should be adjusted for baseline, this can be achieved by removing the treatment main effect, *G*, from the design matrix before fitting the model:

$$\left(\begin{array}{ccccc} Int & T_{1} & T_{2} & T_{1}G & T_{2}G\\ 1 & 0 & 0 & 0 & 0\\ 1 & 1 & 0 & -1 & 0\\ 1 & 0 & 1 & 0 & -1\\ 1 & 0 & 0 & 0 & 0\\ 1 & 1 & 0 & 1 & 0\\ 1 & 0 & 1 & 0 & 1\\ \vdots & \vdots & \vdots & \vdots & \vdots \end{array}\right)\times \left(\begin{array}{ccc} b_{01} & \cdots & b_{0J}\\ b_{11} & \cdots & b_{1J}\\ b_{21} & \cdots & b_{2J}\\ b_{31} & \cdots & b_{3J}\\ b_{41} & \cdots & b_{4J} \end{array}\right)={M_{0}+M}_{T}+M_{T*G}$$

When the main effect for treatment, *G*, is omitted from the model matrix *X*, the treatment effect described by *M*_T*G_ will be adjusted for baseline.

It is also possible to use reference coding for both the time and treatment factor:

$$\left(\begin{array}{cccccc} Int & T_{1} & T_{2} & G & T_{1}G & T_{2}G\\ 1 & 0 & 0 & 0 & 0 & 0\\ 1 & 1 & 0 & 0 & 0 & 0\\ 1 & 0 & 1 & 0 & 0 & 0\\ 1 & 0 & 0 & 1 & 0 & 0\\ 1 & 1 & 0 & 1 & 1 & 0\\ 1 & 0 & 1 & 1 & 0 & 1\\ \vdots & \vdots & \vdots & \vdots & \vdots & \vdots \end{array}\right)\times \left(\begin{array}{ccc} b_{01} & \cdots & b_{0J}\\ b_{11} & \cdots & b_{1J}\\ b_{21} & \cdots & b_{2J}\\ b_{31} & \cdots & b_{3J}\\ b_{41} & \cdots & b_{4J}\\ b_{51} & \cdots & b_{5J} \end{array}\right)={M_{0}+M}_{T}+M_{G}+M_{T*G}$$

If this coding is used, the time effect will change from describing the overall time effect, to only describing the time effect of the reference group. The treatment effect will then be expressed as deviations from the trajectory of the reference group. These two approaches (i.e. using either sum or reference coding for the treatment effect) are related to two earlier methods, known as scaled-to-maximum, aligned, and reduced trajectories (SMART)-analysis [22], and principal response curves (PRC) [23], which involve expressing temporal trajectories relative to a baseline timepoint, or relative to a control group, respectively. Analyzing *M*_*T*_ + *M*_T*G_ is similar to SMART, whereas if we reference code both the time and treatment factors, and then analyze *M*_*G*_ + *M*_T*G_, the result will be similar to PRC. However, neither SMART nor PRC allow inclusion of random effects or baseline adjustments, whereas both are possible in our framework.

Using reference coding for the time factor has implications for the orthogonality of the effect matrices. Because reference coding does not result in orthogonal contrasts, the effect matrices estimated using the repeated measures model are also not mutually orthogonal. Hence, the variance decomposition method commonly used in ASCA is not possible here. However, the interpretation of the effect matrices is still meaningfully defined, as discussed in the previous paragraph. It should also be noted that whenever continuous covariates are included in the model, the design will generally not be fully orthogonal, as there will always be imbalances in the covariate levels between the groups. Hence, the main goal of this approach is not to precisely quantify and decompose mutually independent sources of variation, but rather to estimate and visualize time-varying multivariate treatment effects with improved precision, by extending covariate adjustment strategies used in RCTs to the multivariate case.

So far the random effects structure in the model is only used when estimating the fixed effects in *B*. However, the random effects themselves can also be included and visualized in various ways. In ASCA+/LiMM-PCA the effect matrices are often augmented with the model residuals in order to visualize residual variability in the score plots. For example, if we apply PCA to the time effect matrix, so that *M*_*T*_ = *T*_*T*_*P*_*T*_′, the augmented score matrix is calculated as $T_{T}^{a}=(M_{T}+E)\times P_{T}$ [24]. This can be used to assess the size of the effect compared with the unexplained variation, thereby providing an indirect and qualitative measure of statistical significance. Similarly, we can also augment the effect matrices with the random effects, (*ZU*+*E*), which can be used to visualize the individual offsets (*ZU*), as well as residual variability in response over time (*E*). This can for example be done by applying PCA to the combined effect matrices for the time- and time*treatment interaction effect matrices, so that (*M*_*T*_ + *M*_T*G_) = *T*_*T*+T*G_*P*_*T*+T*G_′, and then calculating the augmented score matrix as $T_{T+T*G}^{a}=(M_{T}+M_{T*G}+ZU+E)\times P_{T+T*G}$. If no other covariates have been included, this becomes equivalent to projecting the raw values onto the components estimated for (*M*_*T*_ + *M*_T*G_). Alternatively, it is also possible to analyze the random effects matrix *ZU* and the residual matrix *E* separately. In a repeated measures model with only a random intercept, the random effect matrix *ZU* is essentially the estimated intercepts for each subject, and will therefore give similar results to a direct PCA on the baseline values. Analyzing the residual matrix *E* is useful for discovering patterns unaccounted for by the model, as well as violations of model assumptions.

#### Model validation

For validation we use nonparametric bootstrapping to construct 95% confidence intervals for the score and loadings associated with each of the effect matrices [19]. This involves resampling whole cases, i.e. all rows in *X*, *Z*, and *Y* belonging to the same subject, until the bootstrap sample reaches the original sample size. We do this within each treatment group separately (i.e. the bootstrapping is stratified by treatment group), which ensures that the number of subjects in each group remains constant across bootstrap samples. The procedure involves first estimating the RM-ASCA+ model from the original data, and collecting the score- and loading estimates from the effect matrices. A bootstrap sample is then created, and the same model is re-estimated. The bootstrapped loading matrices are then rotated towards their corresponding non-bootstrapped loadings using orthogonal Procrustes rotation, and the resulting rotation matrix is then multiplied with the associated bootstrapped score matrix [25,26]. This procedure is repeated a high number of times, e.g. 1000, 10 000, or higher, and the 2.5^th^ and 97.5^th^ percentiles of the bootstrapped score- and loading estimates are used as the lower and upper bounds for the confidence intervals. The bootstrapped effect matrices are mean-centered before PCA during every iteration, in order to focus on variability in the contrast between the levels, rather than their overall magnitude. If scaling is used for the response matrix *Y*, the scaling factors are re-calculated from the bootstrapped data and re-applied during each iteration. This approach only provides an approximate measure of the uncertainty of the estimated mean differences, and should not be interpreted as parametric 95% confidence intervals. However, a simulation study which assessed bootstrapping-based confidence intervals in PRC, suggested that percentile based methods provided generally good coverage at the sample sizes used in this paper [19,27].

### Software and data analysis

All statistical analysis and figure creation were done in MATLAB 2020b. The fitlmematrix-function was used for mixed models, and the pca-function in the Statistics and Machine Learning Toolbox was used for PCA-analysis. MATLAB-code to reproduce the results is available on GitHub at (https://github.com/ntnu-mr-cancer/RM_ASCA). All datasets used in this paper are analyzed using RM-ASCA+ and univariate mixed models. The response variables are scaled to their baseline standard deviation before analysis, to emphasize metabolites with higher variability over time. Mean centering is done prior to PCA on the effect matrices.

## Materials

To demonstrate RM-ASCA+, two published datasets are here used. These will be briefly described, and the reader is directed to their source publications for further details.

### The NeoAva-trial

The first dataset used is from the NeoAva-trial, which is a randomized controlled trial assessing the effect of adding bevacizumab, an anti-angiogenic monoclonal antibody, to conventional chemotherapy in breast cancer patients with locally advanced HER2-negative tumors in a neoadjuvant treatment setting. In a study by Euceda et al., repeated tumor biopsies obtained over the course of treatment were analyzed with high resolution magic angle spinning (HR MAS) MR spectroscopy, using a CPMG sequence [28]. The spectral region between 1.40–4.70 ppm, containing the majority of low-molecular weight metabolites, was selected as the region of interest, and spectral regions containing mostly lipids, ethanol, acetone and lidocaine were excluded. Spectra were PQN-normalized after removal of these areas [29], and metabolites were quantified by peak integration. For further details on spectral acquisition and processing we refer to the original publication by Euceda et al. [28]. Metabolic changes were related to treatment group and tumor response. The published dataset includes 16 quantified and log-transformed metabolites from 122 patients, of whom 60 received bevacizumab + chemotherapy, and 62 received chemotherapy only. Three tumor biopsies were taken; one before start of treatment, one after 12 weeks of treatment, and the last was taken from the surgically removed tumor. Data is missing at all timepoints, with 14%, 36%, and 29% missing outcome data at each timepoint respectively, giving a total of 270 responses in the study.

### Metabolic fingerprint after bariatric surgery

The second dataset used is from a study by Gralka et al., which prospectively assessed alterations in serum metabolites in patients undergoing one of three different kinds of bariatric surgery (proximal Roux-en-Y gastric bypass (RYGB), distal RYGB, and gastric sleeve) [30]. Procedure selection was based on pre-existing clinical factors, such as degree of obesity and comorbidities. Blood was drawn at baseline before surgery, and at 3, 6, 9, and 12 months after surgery, and the serum was analyzed using NMR spectroscopy. Spectra were obtained using a Bruker spectrometer operating at 14.1T, with a triple resonance inverse cryoprobe, and automatic tuning-matching unit and sample changer. A CPMG-sequence was used to acquire the spectra. The water region between 6.0 and 4.5 ppm was removed, and the non-normalized spectra were divided into 0.2 ppm bins, which were integrated using AMIX software (version 3.8.4; Bruker BioSpin). Thirty metabolites were quantified, of which 24 showed variability over time, and 21 were included in the published dataset. The metabolite concentrations were square root transformed prior to analysis to correct for heteroscedasticity. The study design is unbalanced, with 60 patients undergoing distal RYGB, 27 undergoing proximal RYGB, and 19 undergoing gastric sleeve. Data is missing at all post-baseline timepoints, with 7%, 8%, 12%, and 32% missing outcome data at each post-baseline timepoint, respectively, giving a total of 463 responses in the study. The metabolomics data was made freely available by the authors at the online repository MetaboLights, with the identifier MTBLS242.

## Results

### Effect of bevacizumab on tumor metabolism in breast cancer

As the NeoAva study is a randomized controlled trial, a constrained repeated measures model is used to estimate the effect matrices for RM-ASCA+. The following model was used:

$$y_{ihk}=(\beta_{0}+\gamma_{0i})+\beta_{1}T_{1}+\beta_{2}T_{2}+\beta_{3}(T_{1}*Bevacizumab)+\beta_{4}(T_{2}*Bevacizumab)+e_{ihk}$$

where the time factor was reference coded with baseline (*T*_*0*_) as reference, and the variable Bevacizumab was reference coded, where 1 indicates bevacizumab + conventional chemotherapy (Treatment), and 0 indicates conventional chemotherapy only (Control). In order to visualize how the treatment modified the metabolic changes during chemotherapy, the effect matrices for time and the time*treatment interaction factor were added together, and the combined effect matrix was analyzed with PCA. While this confounds the variation from the time- and treatment factors, it also facilitates a more direct assessment of how the treatment and control groups differ at the different timepoints. Results for the time factor and time*treatment factor in isolation are shown in S1 and S2 Figs, respectively. Results from univariate mixed models are shown in S1 Table.

**Fig 1:**
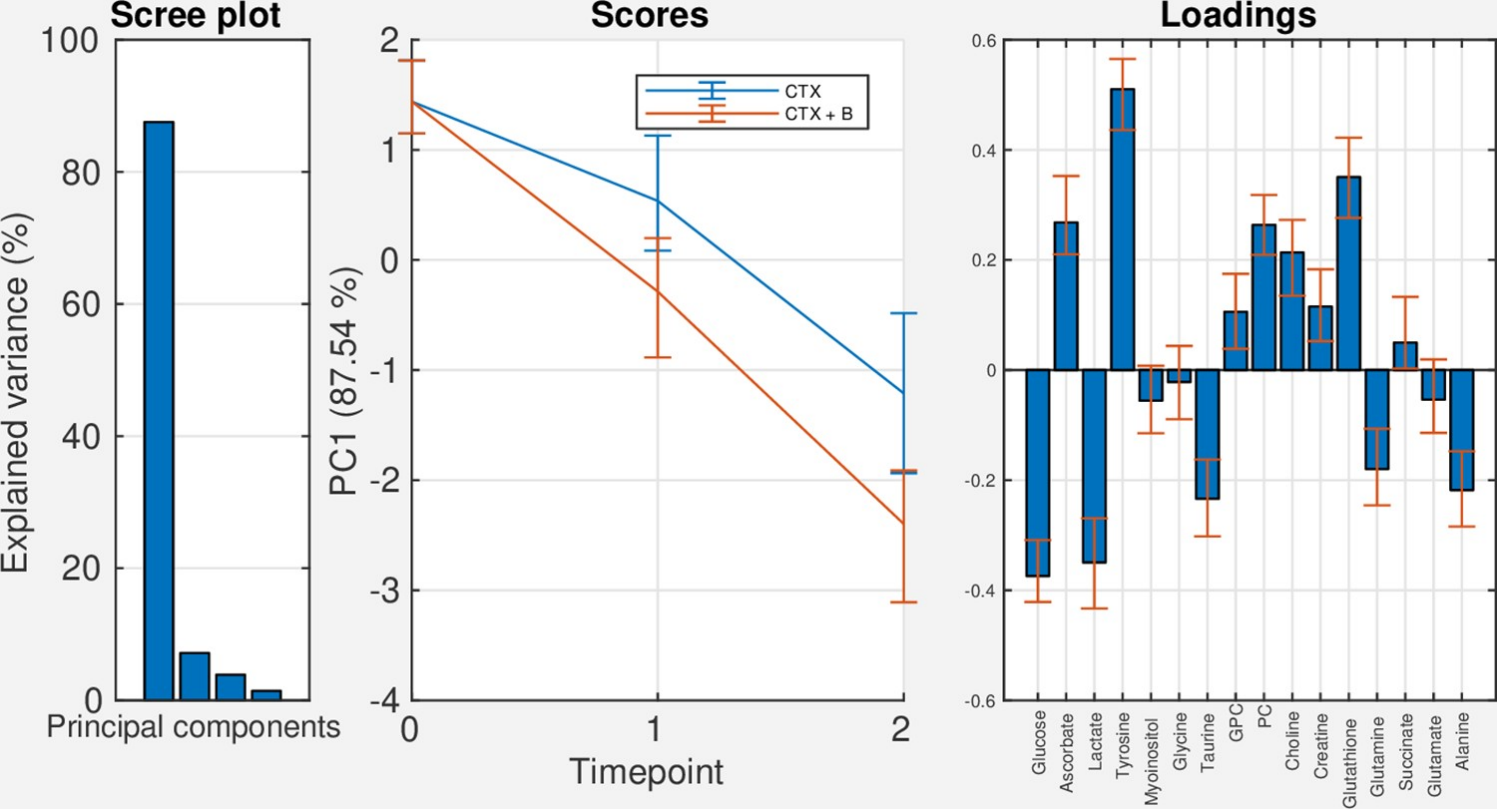
Scree-, score-, and loading plots for the full effect matrix for time + time*treatment. Abbreviations: CTX: Chemotherapy, B: Bevacizumab, GPC: Glycerophosphocholine, PC: Phosphocholine.

In Fig 1, the scree plot shows that the first principal component (PC1) explains approximately 88% of the variation in the time and time*treatment interaction effects. The scores and loadings for PC1 show that levels of ascorbate, tyrosine, glycerophosphocholine, phosphocholine, choline, creatine and glutathione decrease over time, while levels of glucose, lactate, taurine, glutamine, and alanine increase over time, and that these changes are most rapid and pronounced for the treatment group. The bootstrapped confidence intervals suggest a significant time effect at both the second and third timepoints (Figs 1 and S1). No significant treatment effect is observed at the second timepoint, while a marginally significant treatment effect is observed at the third timepoint (Figs 1 and S2).

**Fig 2:**
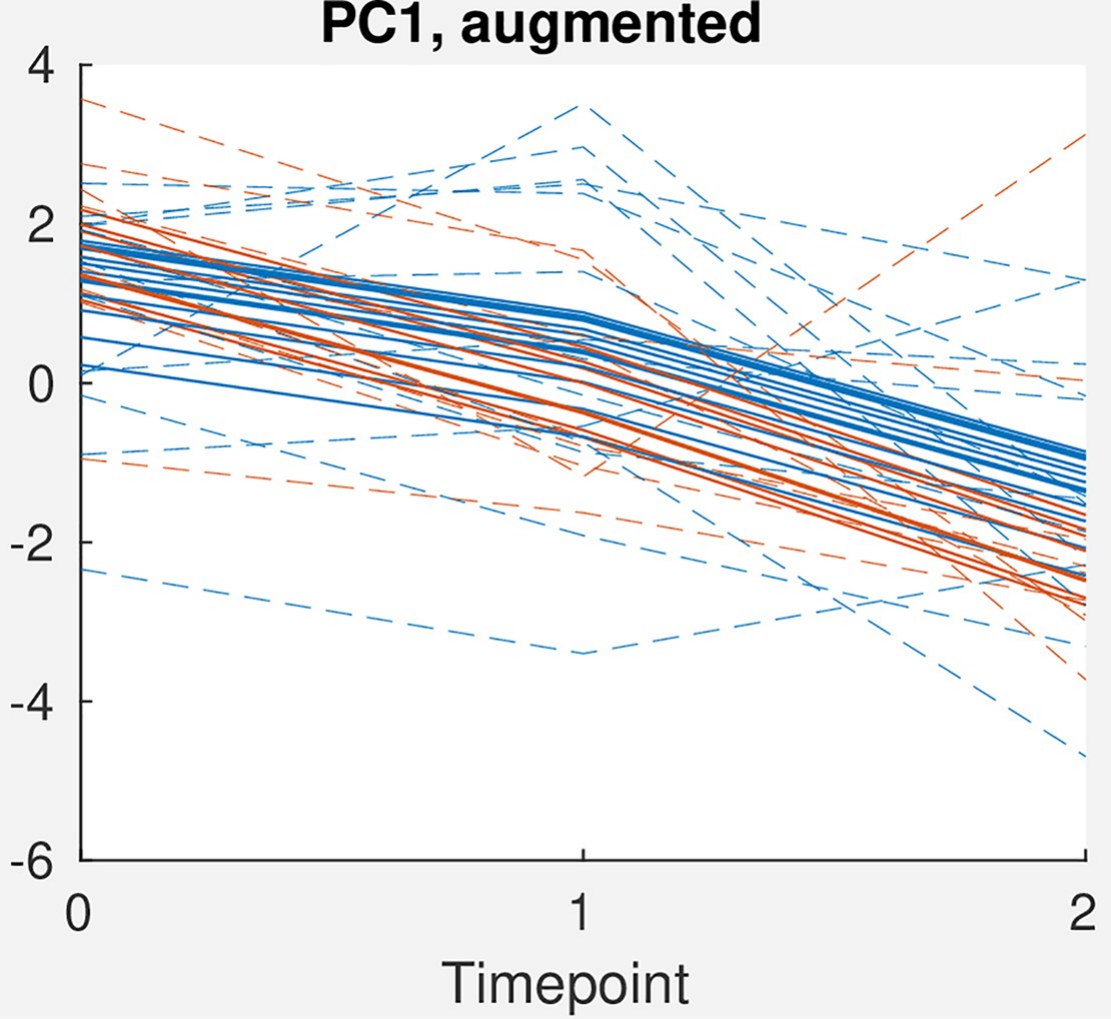
Score plot for PC1 for the effect matrix for time + time*treatment, augmented with random effects (continuous lines) and both random effects and residuals (dashed lines). Only a random subset of patients with complete data are included in the plot. Orange: Chemotherapy + Bevacizumab. Blue: Chemotherapy only.

When the effect matrix for time + time*treatment is augmented with the random intercepts and residuals (Fig 2), we observe significant between-patient variation and heterogeneity in treatment response, showing that the estimated treatment effect is modest relative to the variation between subjects and unexplained variation in the model. For comparison, we have projected both the fitted values (*M*_*T*_ + *M*_T*Bevacizumab_ + *ZU*, continuous lines) and with the residuals added (*M*_*T*_ + *M*_T*Bevacizumab_ + *ZU* + *E*, dashed lines), showing that there is significant residual variation after accounting for the between-subject variation.

### Metabolic effects of bariatric surgery

To estimate the effects for RM-ASCA+ analysis of the bariatric surgery data, an unconstrained repeated measures model was used:

$$y_{ihk}=(\beta_{0}+\gamma_{0i})+\beta_{1}T_{1}+\beta_{2}T_{2}+\beta_{3}T_{3}+\beta_{4}T_{4}+\beta_{5}(Distal)+\beta_{6}(Proximal)+\beta_{7}(Distal*T_{1})+\beta_{8}(Distal*T_{2})+\beta_{9}(Distal*T_{3})+\beta_{10}(Distal*T_{4})+\beta_{11}(Proximal*T_{1})+\beta_{12}(Proximal*T_{2})+\beta_{13}(Proximal*T_{3})+\beta_{14}(Proximal*T_{4})+e_{ihk}$$

where time was reference coded with baseline as reference, and variables for treatment (*Distal* and *Proximal*) were sum coded, with the third category (*Sleeve*) specified by setting both *Distal* and *Proximal* equal to -1. In this analysis the effect matrix for the time factor and the effect matrix for the main effect for treatment together with its interaction with time are analyzed separately. This is done to better isolate the time effect, as the interaction has a large impact on the loadings in the combined analysis. To make the effect matrix for time, a matrix containing the coefficients *β*_*1*_*-β*_*4*_ for each metabolite was multiplied with their corresponding columns in the design matrix *X*. To make the effect matrix for (treatment + time*treatment), the same was done for the coefficients *β*_*5*_*-β*_*14*._ The result from the combined effect matrix is shown in S3 Fig. Results from univariate mixed models are shown in S2 Table.

**Fig 3:**
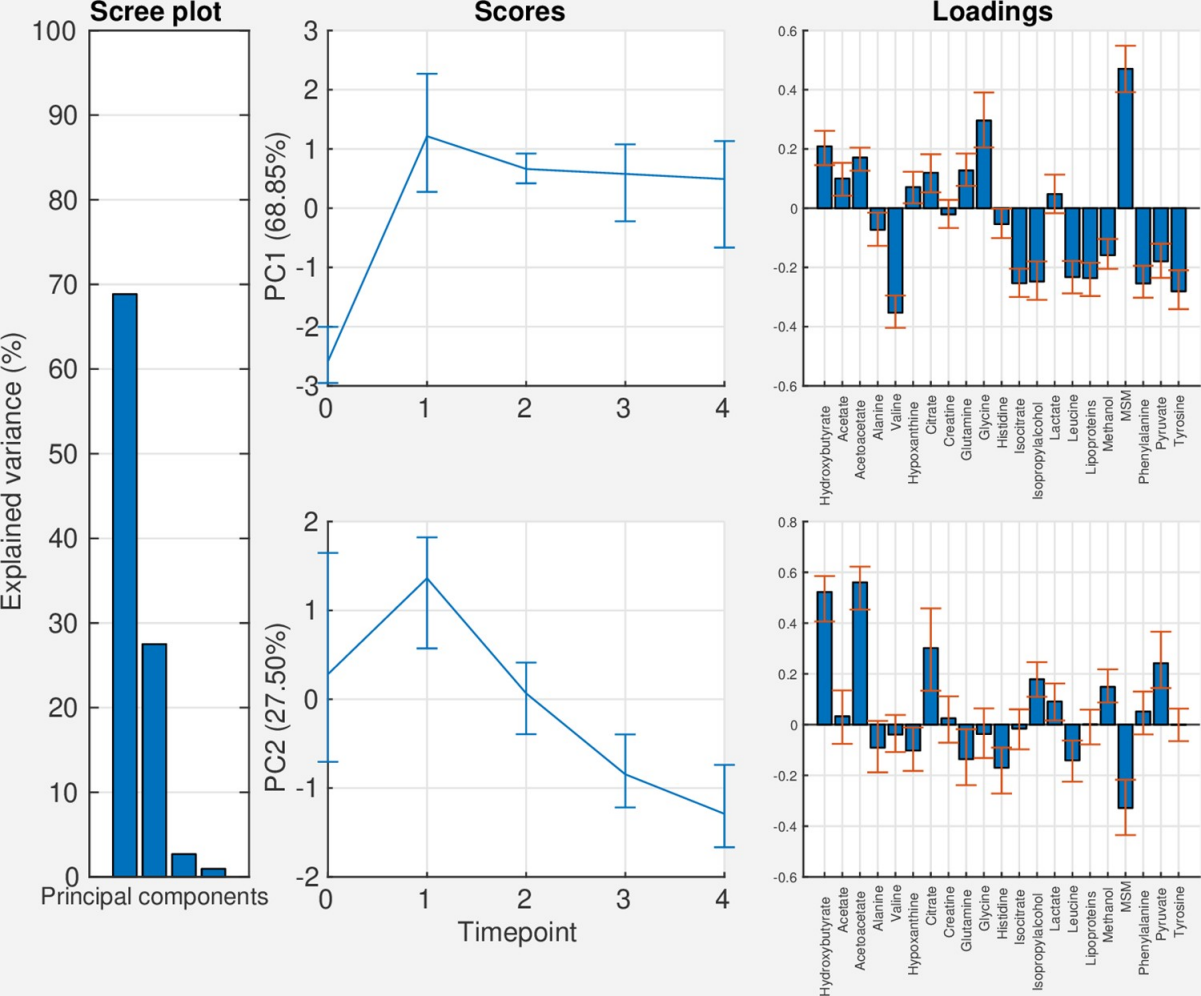
Scree-, score-, and loading plots for the effect matrix for time in the bariatric surgery data. Abbreviations: PC: principal component, MSM: methylsulfonylmethane.

The results from PCA on the effect matrix for time is shown in Fig 3. This trend represents the average change over time across all three groups. Two distinct temporal patterns are observed in the score plots. Along the first component, which explains 69% of the variance in the time effect, there is a highly significant increase in score value between the first and second timepoint, and this difference persists over time. Metabolites with positive loadings on PC1 include methylsulfonylmethane, and the amino acid glycine, while the amino acids valine, isoleucine, tyrosine, and phenylalanine, the alcohols isopropylalcohol and methanol, and the lipoprotein signal have negative loadings. In the second component, which explains 28% of the variance in the time effect, a different pattern is observed. There is a temporary increase in scores after surgery, and then a progressive decrease over time. Metabolites with positive loadings on PC2 mainly include citrate and the ketone bodies acetoacetate and hydroxybutyrate, while methylsulfonylmethane has the most negative loading value.

**Fig 4:**
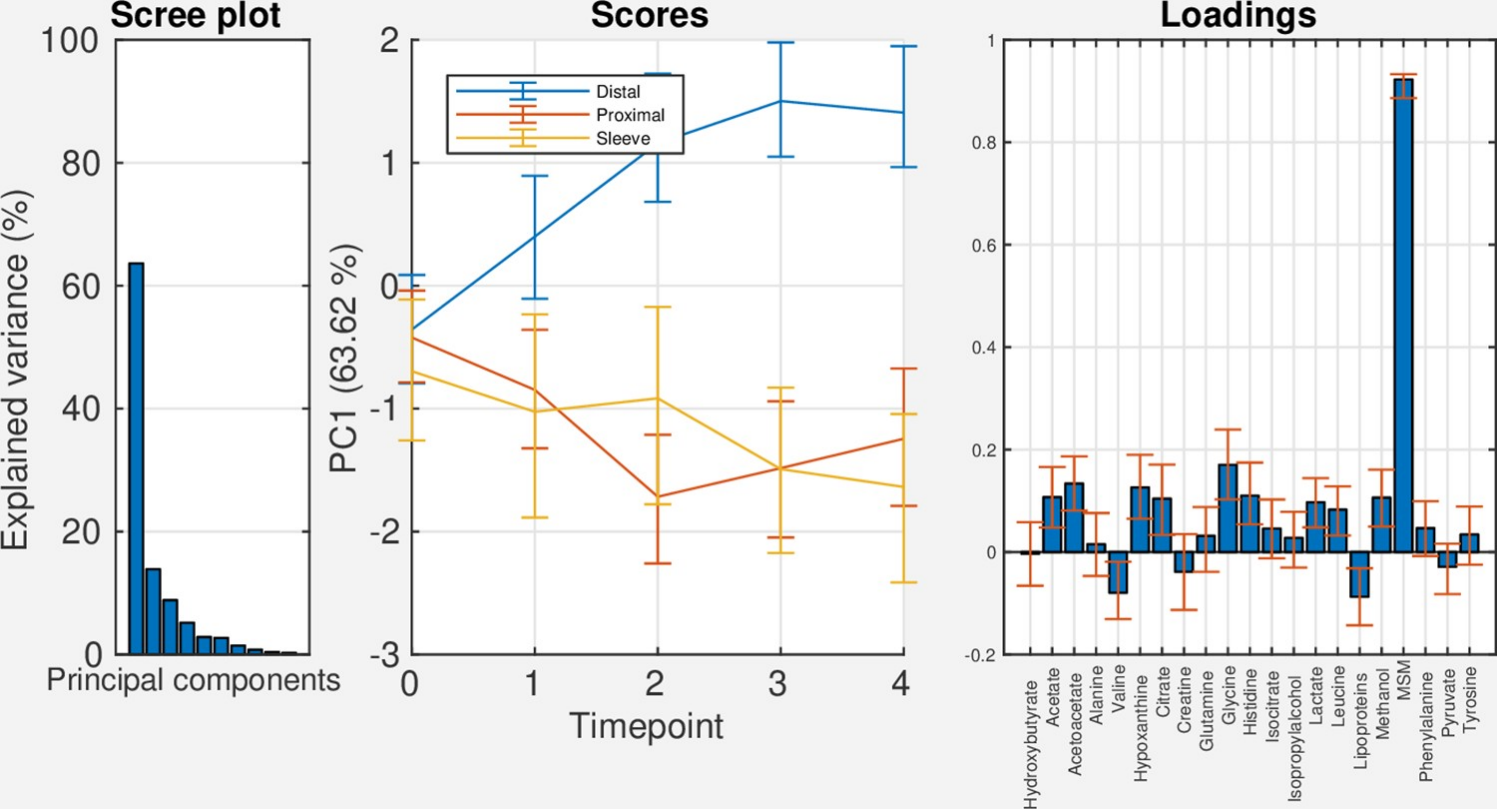
Scree-, score-, and loading plots for the effect matrix for the treatment + time*treatment interaction effect. Abbreviations: PC: principal component, MSM: methylsulfonylmethane.

The results from PCA on the effect matrix for the treatment + time*treatment interaction effect are shown in Fig 4. The first principal component explains 64% of the variation in the effects. In the score plot for PC1, the group receiving distal RYGB shows increasing score values over time, and diverges from the two other surgery groups. The loading plot for PC1 is characterized by a highly positive loading for methylsulfonylmethane.

## Discussion

In this work we have described a novel methodology, RM-ASCA+, suitable for analysis of longitudinal multivariate data, and we have demonstrated this using two publicly available metabolomics datasets. We find that RM-ASCA+ yields interpretable and efficient representations of the findings in the original studies, while also revealing trends not previously apparent. RM-ASCA+ provides a highly flexible methodology that allows different coding systems and inclusion of covariates, making the method highly suitable for clinical trials with multivariate outcomes.

To demonstrate RM-ASCA+, we have used published data from two different clinical trials. In the first study, by Euceda et al., the impact of neoadjuvant bevacizumab on tumor metabolism was assessed in a randomized controlled trial [28]. The statistical analysis in the published paper involved a combination of PCA, PLS-DA, and univariate mixed models. A clear overall metabolic change over time for the entire cohort was described, characterized by increased levels of glucose and lipids, and decreased levels of phosphocholine, glycerophosphocholine, choline, and taurine, which was interpreted as signs of normalization of breast tissue metabolism [31]. However, no significant discrimination between treatment and control was found at any timepoint by PLS-DA. When applying RM-ASCA+, we are able to visualize the fact that while the groups show directionally similar metabolic trajectories over time, the slope is more steep for the treatment group, suggesting that the addition of bevacizumab may have augmented the chemotherapy response.

In the second dataset included in this paper, Gralka et al. assessed metabolic changes in serum after bariatric surgery. In their paper they describe increased levels of the amino acids glycine, glutamine, histidine, and arginine, along with increased levels of methylsulfonylmethane, trimethylamine-N-oxide, and formate, irrespective of procedure type. Conversely, concentrations of the branched chain amino acids (BCAA) isoleucine, leucine, valine, and the aromatic amino acids (AAA) phenylalanine and tyrosine were found to decrease, along with the lipoprotein signal and the gut microbiome derived metabolites methanol and isopropylalcohol. They also describe temporarily increased levels of the ketone bodies acetoacetate and hydroxybutyrate, and citrate after surgery, which was interpreted as reflective of ongoing fat catabolism. We find that by using RM-ASCA+, and then analyzing the time effect matrix, these results are visualized as two separate temporal trends. PC1 shows a large increase in score value from baseline to the first follow-up, and this score remains largely unaltered over time. This component describes the decreased levels of BCAAs, AAAs, lipoprotein signal, and methanol and isopropylalcohol, and increased levels of methylsulfonylmethane, glycine and ketone bodies. The response pattern described by PC2 is characterized primarily by temporarily increased levels of ketone bodies and citrate. These metabolites increase after surgery, before decreasing over time and appearing to approach a steady state, which is not yet reached at the final follow-up. Both the temporal development and metabolite loadings of PC2 suggest that this component may reflect changes in fat catabolism, which presumably is highest in the first months after surgery, and then tapers off as the patients lose weight and reach a new energy equilibrium. Ketone bodies generally increase during periods of increased fat oxidation, due to increased availability of acetyl-CoA, which then react to produce ketone bodies [32]. Analysis of the time-treatment interaction effect showed that the metabolic effects of proximal RYGB and GS were similar, while the group receiving distal RYGB showed a different metabolic trajectory. The loading plots show that this divergence is mainly driven by different effects on the metabolite methylsulfonylmethane, where the group receiving distal RYGB showed more pronounced increases compared with proximal RYGB or GS.

We have discussed three commonly used types of longitudinal linear models: 1) repeated measures models (cLDA/ucLDA), 2) longitudinal ANCOVA, and 3) analysis of changes. While the model types will in some settings yield equivalent results, and all will give unbiased estimates of the treatment effect for randomized studies [33], repeated measures models have some general advantages which make them more suitable for ASCA-type models. In addition to including the baseline responses in the effect matrices, which is useful when visualizing the effects over time in score plots, they can also accommodate both randomized and non-randomized study designs. While longitudinal ANCOVA and analysis of changes also can estimate the time effect, the multivariate results generated using these models are somewhat more difficult to interpret, because the baseline responses are not included in the effect matrices. Repeated measures models also make full use of all available data. For these reasons, we find repeated measures models to be the most suitable for analyzing such studies using ASCA+ or LiMM-PCA.

While LDA-models use all available data when estimating the effects, they require missing data to be missing at random (MAR) in order to provide unbiased estimates. This means that the probability of missingness must be conditionally independent of the value of the unobserved response, given the subject’s observed covariates [34]. For example, if missing samples are metabolically different from non-missing samples from patients with the same covariates, missing data will bias the effect estimates regardless of which linear model is used. This is referred to as missing not at random (MNAR), or non-ignorable missing data. If there is substantial missing data, and it is suspected to be non-ignorable, it is recommended to perform multiple imputation to assess how different assumed distributions for the missing data affect the findings [35]. This can also be implemented in our framework. One relevant example in the context of clinical trials is reference-based imputation [36], where missing responses are imputed under the assumption that their distribution is the same as the observed distribution as one of the treatment arms. If the control group is set as the reference group, this is equivalent to an assumption of no treatment effect for the missing responses. This will lead to a more conservative treatment effect estimate, and may be preferable in cases where MAR is unlikely to hold, and an intention-to-treat analysis is desirable.

An important goal of statistical modeling in clinical trials is to assess whether the treatment effect is statistically significant. In ASCA, the statistical significance of model factors is commonly assessed by permutation testing [37]. However, permutation tests are only approximate for interaction effects [38]. Another approach is to use resampling techniques (e.g. bootstrapping) to generate confidence intervals for the scores and loadings associated with the effect matrices, which is the approach taken in this paper. While this does not provide formal statistical inference or exact p-values, bootstrap-based confidence intervals have been explored for several related methods, including multilevel simultaneous component analysis (ML-SCA) [27], which can be considered a special case of ASCA, and PRC [19], and have been found to perform reasonably well provided a sufficient sample size. Because principal components based on different data are not necessarily comparable due to rotational ambiguity, we also performed a rotation step before obtaining the percentiles [25,26]. The rotation was done using the loadings as the target matrix, and this naturally results in more narrow confidence intervals for the loadings. While this approach has the advantage of providing confidence intervals for both scores and loadings, the exact interpretation of these intervals has still not been properly studied, and further investigation into their inferential properties is still needed. An alternative way of assessing statistical significance of experimental factors is provided by the GLLR-test in the LiMM-PCA method. Given that the main aim of clinical trials is to estimate and test the effect of treatments, further research should be done to systematically compare the different methods for assessing statistical significance in the ASCA+ / LiMM-PCA frameworks in the context of randomized controlled trials.

A limitation of RM-ASCA+ is that mixed models are computationally demanding, and applying mixed models to potentially tens of thousands of variables may not be feasible. In such situations, the pre-transformation of the response matrix by PCA done in LiMM-PCA can drastically reduce the number of response variables, making LiMM-PCA the more scalable alternative for high-dimensional data. Another issue is the impact of pre-processing. Both scaling and normalization methods, as well as variable transformations such as log- or square root transformations, can strongly impact multivariate models, including ASCA [21]. A discussion of the impact of these issues is outside the scope of this paper, but they should be carefully considered in any multivariate analysis study. Finally, there is also the possibility of extending this methodology to count and binary data using generalized linear mixed models. This extension may be problematic for LiMM-PCA, because the initial PCA-step may not be well suited for these data types, while this is less of an issue in RM-ASCA+, which does not include this step.

In conclusion, repeated measures linear mixed models can be used to visualize and compare multivariate changes between groups over time. This approach is not limited to metabolomics data, but may be suitable for any study using a longitudinal repeated measures design with a multivariate endpoint.

## Supporting information

S1 Fig Scree-, score-, and loading plots for the effect matrix for time for the NeoAva-data. Abbreviations: CTX: Chemotherapy, B: Bevacizumab, GPC: Glycerophosphocholine, PC: Phosphocholine.
(TIF)

S2 Fig Scree-, score-, and loading plots for the effect matrix for time*treatment interaction for the NeoAva-data. Abbreviations: CTX: Chemotherapy, B: Bevacizumab, GPC: Glycerophosphocholine, PC: Phosphocholine.
(TIF)

S3 Fig Scree-, score-, and loading plots for the effect matrix for time + treatment + time*treatment interaction for the bariatric surgery data. Abbreviations: PC: principal component, MSM: methylsulfonylmethane.
(TIF)

S1 Table Table with results from analyses with individual linear mixed models (constrained LDA-model) on each metabolite for the NeoAva-data. (XLSX)

S2 Table Table with results from analyses with individual linear mixed models (unconstrained LDA-model) on each metabolite for the bariatric surgery data. (XLSX)

## References

[1] Smilde AK, Timmerman ME, Hendriks MM, Jansen JJ, Hoefsloot HC. Generic framework for high-dimensional fixed-effects ANOVA. Brief Bioinform. 2012;13(5):524–35. doi: 10.1093/bib/bbr071 22199378

[2] Smilde AK, Jansen JJ, Hoefsloot HC, Lamers RJ, van der Greef J, Timmerman ME. ANOVA-simultaneous component analysis (ASCA): a new tool for analyzing designed metabolomics data. Bioinformatics. 2005;21(13):3043–8. doi: 10.1093/bioinformatics/bti476 15890747

[3] Thiel M, Féraud B, Govaerts B. ASCA+ and APCA+: Extensions of ASCA and APCA in the analysis of unbalanced multifactorial designs. Journal of Chemometrics. 2017;31(6):e2895.

[4] Martin M, Govaerts B. LiMM-PCA: Combining ASCA+ and linear mixed models to analyse high-dimensional designed data. Journal of Chemometrics. 2020;34(6):e3232.

[5] Wang J, Reijmers T, Chen L, Van Der Heijden R, Wang M, Peng S, et al. Systems toxicology study of doxorubicin on rats using ultra performance liquid chromatography coupled with mass spectrometry based metabolomics. Metabolomics. 2009;5(4):407–18. doi: 10.1007/s11306-009-0165-3 20046867 PMC2794350

[6] Detry MA, Ma Y. Analyzing Repeated Measurements Using Mixed Models. JAMA. 2016;315(4):407–8. doi: 10.1001/jama.2015.19394 26813213

[7] Pearl J. Lord’s Paradox Revisited–(Oh Lord! Kumbaya!). Journal of Causal Inference. 2016;4(2):20160021.

[8] J T, L B, T H, J R, M W, M H. Different ways to estimate treatment effects in randomised controlled trials. Contemporary Clinical Trials Communications. 2018;10:80–5. doi: 10.1016/j.conctc.2018.03.008 29696162 PMC5898524

[9] Lu K, Mehrotra DV, Liu G. Sample size determination for constrained longitudinal data analysis. Statistics in Medicine. 2009;28(4):679–99. doi: 10.1002/sim.3507 19051207

[10] Van Breukelen GJ. ANCOVA versus change from baseline: more power in randomized studies, more bias in nonrandomized studies [corrected]. J Clin Epidemiol. 2006;59(9):920–5. doi: 10.1016/j.jclinepi.2006.02.007 16895814

[11] van Breukelen GJP. ANCOVA Versus CHANGE From Baseline in Nonrandomized Studies: The Difference. Multivariate Behavioral Research. 2013;48(6):895–922. doi: 10.1080/00273171.2013.831743 26745598

[12] Austin PC, Manca A, Zwarenstein M, Juurlink DN, Stanbrook MB. A substantial and confusing variation exists in handling of baseline covariates in randomized controlled trials: a review of trials published in leading medical journals. Journal of Clinical Epidemiology. 2010;63(2):142–53. doi: 10.1016/j.jclinepi.2009.06.002 19716262

[13] Austin PC, Manca A, Zwarenstein M, Juurlink DN, Stanbrook MB. Covariate adjustment in RCTs results in increased power to detect conditional effects compared with the power to detect unadjusted or marginal effects. Journal of Clinical Epidemiology. 2010;63(12):1392–3.

[14] Kahan BC, Morris TP. Reporting and analysis of trials using stratified randomisation in leading medical journals: review and reanalysis. BMJ: British Medical Journal. 2012;345:e5840. doi: 10.1136/bmj.e5840 22983531 PMC3444136

[15] Kahan BC, Morris TP. Improper analysis of trials randomised using stratified blocks or minimisation. Statistics in Medicine. 2012;31(4):328–40. doi: 10.1002/sim.4431 22139891

[16] Lord FM. A paradox in the interpretation of group comparisons. Psychol Bull. 1967;68(5):304–5. doi: 10.1037/h0025105 6062585

[17] Glymour MM, Weuve J, Berkman LF, Kawachi I, Robins JM. When Is Baseline Adjustment Useful in Analyses of Change? An Example with Education and Cognitive Change. American journal of epidemiology. 2005;162(3):267–78. doi: 10.1093/aje/kwi187 15987729

[18] Conesa A, Nueda MJ, Ferrer A, Talón M. maSigPro: a method to identify significantly differential expression profiles in time-course microarray experiments. Bioinformatics. 2006;22(9):1096–102. doi: 10.1093/bioinformatics/btl056 16481333

[19] Timmerman ME, Ter Braak CJF. Bootstrap confidence intervals for principal response curves. Computational Statistics & Data Analysis. 2008;52(4):1837–49.

[20] Scodes J. Baseline Mean Centering for Analysis of Covariance (ANCOVA) Method of Randomized Controlled Trial Data Analysis.

[21] Timmerman ME, Hoefsloot HCJ, Smilde AK, Ceulemans E. Scaling in ANOVA-simultaneous component analysis. Metabolomics. 2015;11(5):1265–76. doi: 10.1007/s11306-015-0785-8 26366136 PMC4559107

[22] Keun HC, Ebbels TMD, Bollard ME, Beckonert O, Antti H, Holmes E, et al. Geometric Trajectory Analysis of Metabolic Responses To Toxicity Can Define Treatment Specific Profiles. Chemical Research in Toxicology. 2004;17(5):579–87. doi: 10.1021/tx034212w 15144214

[23] Van den Brink PJ, Braak CJFT. Principal response curves: Analysis of time-dependent multivariate responses of biological community to stress. Environmental Toxicology and Chemistry. 1999;18(2):138–48.

[24] Zwanenburg G, Hoefsloot HCJ, Westerhuis JA, Jansen JJ, Smilde AK. ANOVA–principal component analysis and ANOVA–simultaneous component analysis: a comparison. Journal of Chemometrics. 2011;25(10):561–7.

[25] Martens H, Martens M. Modified Jack-knife estimation of parameter uncertainty in bilinear modelling by partial least squares regression (PLSR). Food Quality and Preference. 2000;11(1):5–16.

[26] Timmerman ME, Kiers HA, Smilde AK. Estimating confidence intervals for principal component loadings: a comparison between the bootstrap and asymptotic results. Br J Math Stat Psychol. 2007;60(Pt 2):295–314. doi: 10.1348/000711006X109636 17971271

[27] Timmerman ME, Kiers HAL, Smilde AK, Ceulemans E, Stouten J. Bootstrap confidence intervals in multi-level simultaneous component analysis. British Journal of Mathematical and Statistical Psychology. 2009;62(2):299–318. doi: 10.1348/000711007X265894 18086338

[28] Euceda LR, Haukaas TH, Giskeødegård GF, Vettukattil R, Engel J, Silwal-Pandit L, et al. Evaluation of metabolomic changes during neoadjuvant chemotherapy combined with bevacizumab in breast cancer using MR spectroscopy. Metabolomics. 2017;13(4):37.

[29] Dieterle F, Ross A, Schlotterbeck G, Senn H. Probabilistic quotient normalization as robust method to account for dilution of complex biological mixtures. Application in 1H NMR metabonomics. Anal Chem. 2006;78(13):4281–90. doi: 10.1021/ac051632c 16808434

[30] Gralka E, Luchinat C, Tenori L, Ernst B, Thurnheer M, Schultes B. Metabolomic fingerprint of severe obesity is dynamically affected by bariatric surgery in a procedure-dependent manner. Am J Clin Nutr. 2015;102(6):1313–22. doi: 10.3945/ajcn.115.110536 26581381

[31] Vander Heiden MG, Cantley LC, Thompson CB. Understanding the Warburg effect: the metabolic requirements of cell proliferation. Science. 2009;324(5930):1029–33. doi: 10.1126/science.1160809 19460998 PMC2849637

[32] Laffel L. Ketone bodies: a review of physiology, pathophysiology and application of monitoring to diabetes. Diabetes/Metabolism Research and Reviews. 1999;15(6):412–26. doi: 10.1002/(sici)1520-7560(199911/12)15:6&lt;412::aid-dmrr72&gt;3.0.co;2-8 10634967

[33] Senn S. Baseline Adjustment in Longitudinal Studies. Encyclopedia of Biostatistics 2005.

[34] Bhaskaran K, Smeeth L. What is the difference between missing completely at random and missing at random? Int J Epidemiol. 2014;43(4):1336–9. doi: 10.1093/ije/dyu080 24706730 PMC4121561

[35] Jakobsen JC, Gluud C, Wetterslev J, Winkel P. When and how should multiple imputation be used for handling missing data in randomised clinical trials–a practical guide with flowcharts. BMC Medical Research Methodology. 2017;17(1):162. doi: 10.1186/s12874-017-0442-1 29207961 PMC5717805

[36] Liu GF, Pang L. On analysis of longitudinal clinical trials with missing data using reference-based imputation. Journal of Biopharmaceutical Statistics. 2016;26(5):924–36. doi: 10.1080/10543406.2015.1094810 26418282

[37] Vis DJ, Westerhuis JA, Smilde AK, van der Greef J. Statistical validation of megavariate effects in ASCA. BMC Bioinformatics. 2007;8(1):322. doi: 10.1186/1471-2105-8-322 17760983 PMC2211757

[38] Anderson M, Braak CT. Permutation tests for multi-factorial analysis of variance. Journal of Statistical Computation and Simulation. 2003;73(2):85–113.

