# Supplementary figures and images for: Repeated measures ASCA+ for analysis of longitudinal intervention studies with multivariate outcome data
Source: PLoS Comput Biol. 2021 Nov 9;17(11):e1009585. doi: 10.1371/journal.pcbi.1009585 (PMC8604364; doi:10.1371/journal.pcbi.1009585)

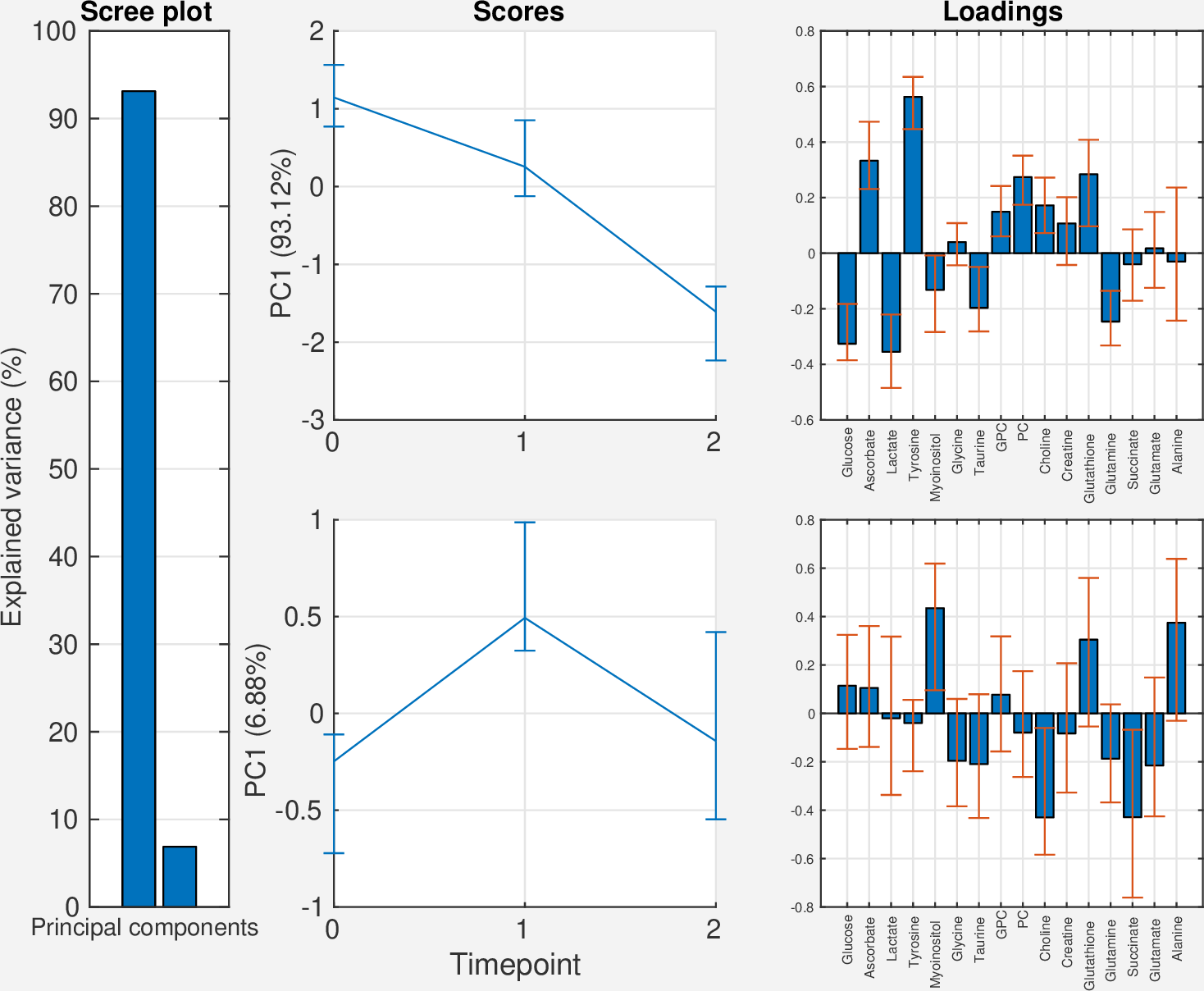

Supplement: S1 Fig — Abbreviations: CTX: Chemotherapy, B: Bevacizumab, GPC: Glycerophosphocholine, PC: Phosphocholine. (TIF) [file pcbi.1009585.s001.tif]

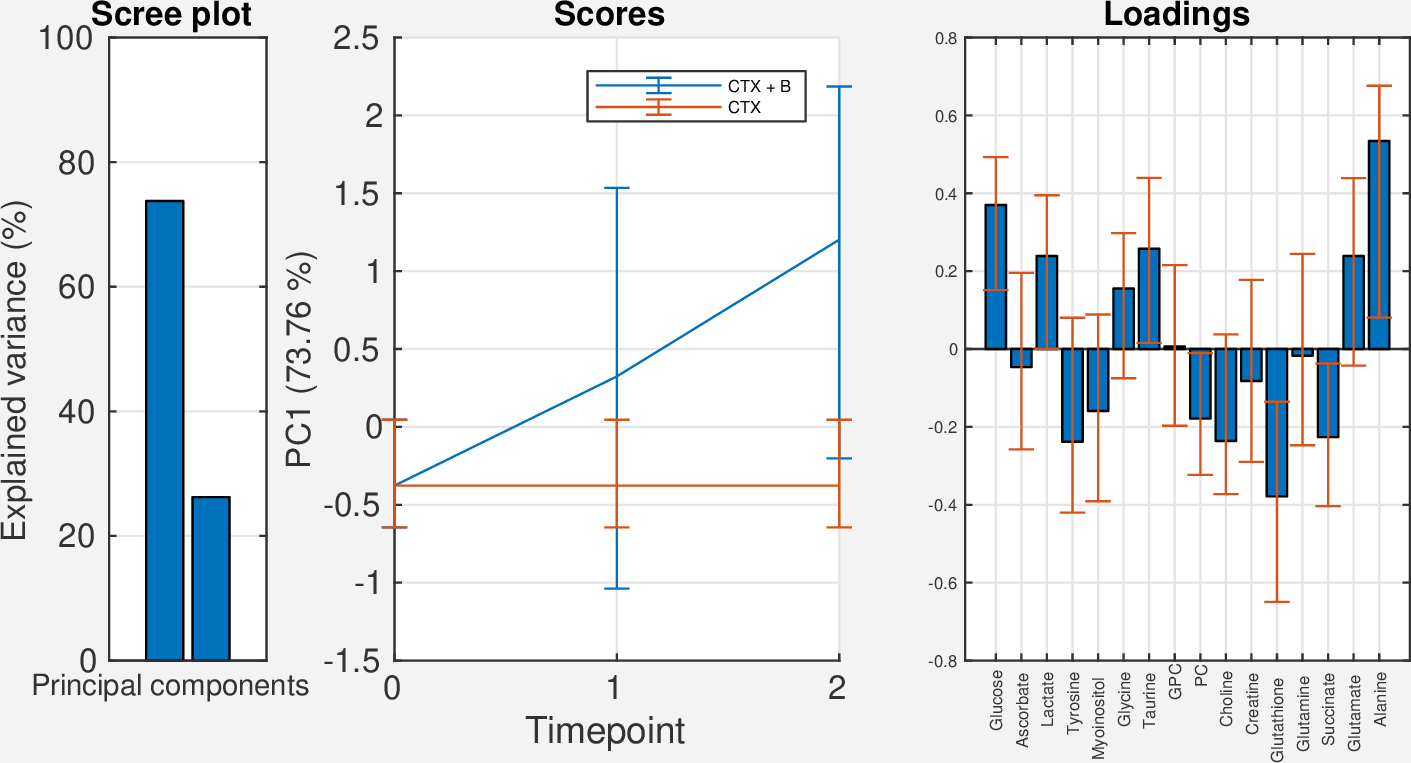

Supplement: S2 Fig — Abbreviations: CTX: Chemotherapy, B: Bevacizumab, GPC: Glycerophosphocholine, PC: Phosphocholine. (TIF) [file pcbi.1009585.s002.tif]

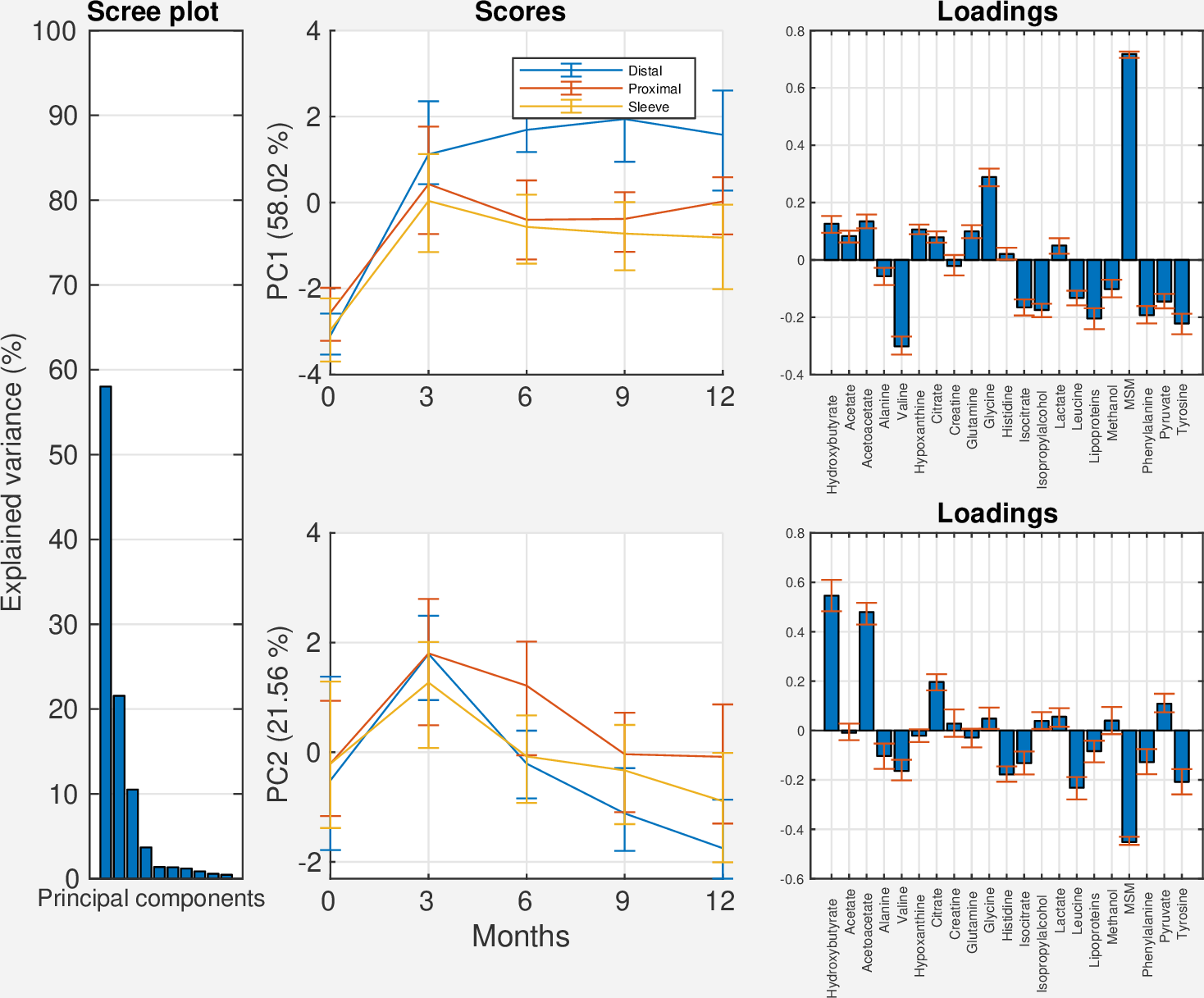

Supplement: S3 Fig — Abbreviations: PC: principal component, MSM: methylsulfonylmethane. (TIF) [file pcbi.1009585.s003.tif]
